# Sero-prevalence of viral hepatitis A in a district of Sri Lanka: a community based cross-sectional study

**DOI:** 10.1186/s12879-019-4043-y

**Published:** 2019-05-21

**Authors:** Nalin Ariyarathna, Chrishantha Abeysena

**Affiliations:** 1 Regional Director of Health Services Office, Ministry of Health, Gampaha, Sri Lanka; 2 Department of Public Health, Faculty of Medicine, University of Kelaniya, Kelaniya, Sri Lanka

**Keywords:** Community, Hepatitis, Sero-prevalence, Viral

## Abstract

**Background:**

Hepatitis A Virus (HAV) is one of the most common food and water borne infectious disease prevailing globally. The objective of the study was to determine sero-prevalence of HAV infection in a district of Sri Lanka.

**Methods:**

This was a descriptive cross sectional study conducted on 1403 participants aged 1 year and above selected by multistage stratified (for age group and area of residence) cluster sampling from September 2015 to December, 2016. An interviewer-administered questionnaire was used to collect data and Anti-IgG testing was done to determine sero-positivity. The overall, the age and sex specific sero-prevalence of HAV were calculated with 95% confidence intervals (CI).

**Results:**

Of the 1403 participants 1132 were anti HAV IgG positive. Therefore the overall sero-prevalence of HAV infection was 80.7% (95%CI: 78.64–82.76). There were 283 (20.2%) individuals below the age group of 14 years and below and out of them, 204 had anti HAV IgG, therefore sero-prevalence was 72.1% for that age group. The age group 15 years and aboe comprised of 1120 (79.8%) participants and of them 928 had anti HAV IgG, making sero-prevalence 82.9%. The lowest sero-prevalence (66.9%, *n* = 232) was observed in the age group of 11–20 years followed by 21–30 age group. From age 31 years onwards, the sero-prevalence exceeded 90%, reaching 100% after 71 years. The urban population showed a sero-prevalence of 83% (*n* = 195) and 80.2% (*n* = 937) for the rural sector while females had a sero-prevalence of 82.2% (*n* = 766) and it was 77.7% (*n* = 366) for males. Thirty-four (3.0%) participants who had sero-positive results (*n* = 1132) claimed that they have had HAV in the past.

**Conclusions:**

Overall, four fifth of the population was immune to HAV infection in the district of Gampaha.

## Background

An estimated 1.5 million individuals worldwide are affected by hepatitis A virus (HAV). The HAV infection tends to be asymptomatic or only mildly symptomatic in young children, but the likelihood of experiencing symptomatic disease increases with age [1]. The HAV causes a systemic disease following transmission via the feco-oral route. Viraemia that follows infection gives rise to clinical manifestations of the disease including nausea, anorexia, fever and a general feeling of un-wellness [2]. Patients may also experience several months of debility following resolution of symptoms. Known as ‘post-hepatitis syndrome, this is a functional illness that affects the patient even after the biochemical indicators have returned to normal levels [2].

Prognosis of HAV is usually excellent in young adults with mortality rates as low as 0.1%. For those aged 40 and above, mortality rate can be as high as 2.1% [3]. Rarely HAV can cause extra hepatic complications such as arthritis, vasculitis, myocarditis and renal failure [2]. HAV also leads to a heavy economic burden, resulting from lost productivity due to absenteeism from work as well as through direct medical costs following hospitalization [1]. In the case of high endemic countries, the economy is also affected due to its impact on tourism where foreign visitors may need to be vaccinated before visiting. Occurrences such as these give a negative impression of the country.

Serological diagnosis of HAV infection involves the detection of HAV specific antibodies in the serum of a suspected patient. Anti-HAV IgM is present in recent infection with levels peaking in early infection and lasting for no more than 4–6 months thereafter. Anti-HAV IgG is the antibody responsible for conferring lifelong immunity to those who are infected with the virus. Though, IgG antibodies appear early in the infection, they only peak during the convalescence and can be detected lifelong from then on [3]. In fact most of the general population over the age of 50 years have been found to be positive for anti-HAV antibodies, while in areas of high prevalence, majority of children over the age of 3 years have also been found to be positive for anti-HAV IgG [2]. However in newborns, the disappearance of maternal antibodies is observed to take place around the age of 1 year. Thus antibodies detected after this period can best be considered as their own antibodies [4].

Sero-prevalence of HAV can be defined as the prevalence of hepatitis A antibodies in a given population. Sero-prevalence is also directly related to the endemicity of HAV as well as the susceptibility of individuals to the infection. Therefore determination of sero-prevalence allows assessment of the need for vaccination and to determine the best age group for vaccination.

A study conducted in Cambodia in 1990 revealed that 90% of children and 100% of adults were immune to HAV. Malaysia and Thailand were found to have decreasing trends in sero-prevalence in 1998 while most of Indonesia, Vietnam and the Philippines did not demonstrate such reduction.

In Sri Lanka, the national average incidence of HAV in 2011 was 0.79 per 1000 population. In the same year, the incidence of HAV in the district of Gampaha was 2.06 per 1000 population, which was more than twice of the national average. The District of Gampaha ranked as the 3rd in terms of prevalence of HAV in Sri Lanka in the year 2011 [5]. Furthermore, there were total of 1622, 1697 and 1564 clinically confirmed viral hepatitis incidence cases identified respectively in year 2013, 2014 and 2015 island-wide [6]. This indicates that a high sero-prevalence of HAV could be expected in the district of Gampaha. Firstly Gampaha is largely populated district in the country and being the sixth largest urban area in Western Province the there is a reasonably large urban population but the majority of the population belongs to the rural sector. Secondly, there had been no documented study conducted to determine sero-prevalence of HAV infection in Gampaha. However, the current factual situation of HAV burden in Sri Lanka is not known since there is no recent evidence regarding sero-prevalence. The only available community based study on sero-prevalence was conducted in Colombo district in 1979 [7]. Endemicity of viral Hepatitis A is changing globally and many countries are researching on changes of endemicity in order to focus on preventive strategies especially targeting group immunization [8]. Hence, identifying the current HAV burden in Sri Lanka is a timely need of the country. Therefore the objective of the study was to determine the sero-prevalence of viral hepatitis A in the district of Gampaha in Sri Lanka.

## Methods

A community based descriptive cross sectional study was conducted in the district of Gampaha, Sri Lanka from 1st of September 2015 to 15^th^of December, 2016. The district had a population of 2316, 000 [5]. The study population included individuals of both sexes aged 1 year onwards and who were permanent residents in the District of Gampaha. The exclusion criteria were infants and patients with psychiatric illness.

The sample size calculation was performed by expecting a population proportion and absolute precision as 50 and 4% respectively with 5% significant level for which the critical value is 1.96 for a two sided test. The calculated sample size was 600.To account for the cluster effect, it was necessary to adjust the required sample size having it multiplied by the design effect which was taken as two [9]. A further adjustment to the sample size was made and considering a non-response rate of 15% the required sample size was 1411.

A multi-stage stratified cluster sampling technique was adopted to select the required number of participants. The district of Gampaha consists of 12 Pradesheeyasabhas, five Urban Councils and two Municipal Council areas. There are 1177 Grama-Niladari’ Divisions (GND) in the district of Gampaha and a GND was considered as a cluster. Pradesheeyasabha areas were considered as rural strata while urban councils and municipal areas were considered as urban strata. The population distribution in the rural strata was 1,950,304 (84.31%) in 1059 GND while in urban strata it was 361,497 (15.62%) distributed in 118 GND. Thirty clusters were selected according to the aforesaid proportions and in that manner five GND clusters from urban sector and 25 GND’s from rural sector were included. The cluster size was obtained by the division of 1400 by 30, thus making 48 as the size of one cluster. The next step of stratification was based on ages 01–14 years and 15 years and above. Those aged 14 years and below consisted of 22.8% of the total population of the district of Gampaha (527,434 individuals) while remaining 77.2% fell in to the category of those aged 15 years and above with a population of 1,785,566 [5] allowing a ratio of 1:3 (22.8% vs. 77.2%). Therefore 1112 (77.2%) study participants were selected from the age group 15 years and above and 328 (22.8%) from the age group 14 years and below.

The list of GNDs in the district was obtained for the selection procedure and the original order of GND was not changed in the process of selection of GND clusters. The respective populations and the cumulative population of all GNDs within the rural sector and urban sector were listed separately prior to the selection. Thirty clusters were selected which are probability proportionate to size of the GNDs separately selected in the urban and rural areas.

Selection of households within the cluster was done using voters’ registry (sampling frame). A serial number was given to each household in the registry and the first household was selected from the list by a randomly selected computer generated serial number. Second household was the left closest household of the selected first household. If a left side house was not found, right side closest house was selected. This procedure was done until required numbers of households were selected. If a house was permanently closed that particular house was not considered for the study. Second visits were made in order to select individuals from houses which were closed temporarily at the time of selection procedure. A person or group of people who lived together were considered as a household and they should be permanent residents under one chief house occupant. Only one individual in a particular household was selected to the study. Pre-determined age stratification was a concern when selecting an individual from a household. Thus 12 individuals out of 48 individuals who were 14 years or less, and 36 individuals who were 15 years and above were planned to be selected from each cluster. In the case an individual aged 14 or below was unavailable at the selected household, immediate next household was chosen. One individual aged 15 years and above was recruited from the three out of four eligible houses. This same cyclic process was continued until the desired numbers of individuals were obtained. Within the household, every individual eligible for the study was listed in the ascending order of their ages. One individual was selected randomly from the prepared list of intra household members by drawing lots. Visits were arranged in the weekends and on public holidays to reach maximum number of individual available in households.

Data was collected by a pre-tested interviewer administered questionnaire. This questionnaire was used to obtain socio-demographic data and clinical information including symptoms manifested and the past medical details related to viral hepatitis. The pre-test was carried out with the participation of 20 individuals from the district of Gampaha who were eligible but were not selected as participants of the study. Feedback from the participants was noted and used as a basis for improving the questionnaire.

Two trained two pre-intern medical graduates were selected as interviewers. Written guidelines were given to them following the training to refer whenever they require necessary guidance. Each and every selected household was visited on a weekend in order to find maximum number of household members in a cluster. Selected individuals from households were informed about the purpose of the study and written consent was obtained. In the case of a child aged 18 years or less, consent was obtained from parents or the guardian in addition to assent from the child. Following the completion of the selection process selected individuals were given a convenient date, time and place close to their living premises in order to collect data and blood samples. Sometimes this process had to be repeated twice or more to complete the collection of data within the cluster.

Blood samples were collected by the Public Health Nursing Sister (PHNS) for serological findings. Five milliliters of blood were collected from each individual with adherence to a protocol on collection procedure. Collected samples were transported in a cool box to the laboratory facility at the University of Colombo on the same day. Separated serum was kept in the temperature of minus 20 degrees centigrade in order to preserve serum samples until the tests were conducted. At the end of the collection of blood and data, participants were appreciated and assured of the confidentiality of the data gathered. Completeness and clarity of data was a concern. At least 10% of the completed questionnaires were picked at random and re-checked to ensure validity. Confirmation of HAV is solely based on the laboratory confirmation with presence of antibodies in the serum. Serology testing was performed by using AccuDiag anti-HAV-IgG ELISA kits. The test is 100% sensitive and 99% specific for detecting HAV. The tests were done in the Microbiology laboratory of the Faculty of Medicine, University of Colombo, under the direct supervision of a Microbiologist. Quality control methods were strictly adhered to when performing the tests.

Data were analyzed using Statistical Package for Social Sciences 16. Overall, the age and sex specific sero-prevalence of HAV were calculated with 95% confidence interval (CI).

## Results

Out of 1440 individuals who were initially selected for the study, only 1403 individuals participated. Response rate was 97.4%. Of the participants, 83.3% (*n* = 1168) were from 25 rural GND clusters and 16.7% (*n* = 235) from urban GND clusters.

The mean (SD) age of the participants was 29 (18.7) years. Maximum age was observed as 95 years. Mean (SD) age of males were 25.8 (20.1) years while mean (SD) age of females were 31.3 (17.7) years. Among the participants 66.4% were females (*n* = 932). Out of 1403 majority were Sinhalese (*n* = 1347, 96.01%), rest were Tamils 3.0% (*n* = 42) and Muslims 0.7% (*n* = 10). Among the participants 49.9% (*n* = 700) were unmarried while 48.6% (*n* = 682) were married and the remaining were widowed (1.5%, *n* = 21). Seventy seven point 9 % (*n* = 1093) of the participants were Buddhist while 20.2% (*n* = 284) were Catholics. Majority of the participant had completed their education up to Grade 11 49.3% (*n* = 284) while only 4.4% (*n* = 61) had completed education up to the tertiary level. Among the respondents, majority were unemployed (*n* = 1060, 75.6%) and paid workers were 242, (17.3%) including pensioners. Family income of the majority (*n* = 644, 45.9%) was less than 20,000 followed by the category of 20,000–39,999 LKR. Only 14 (1%) among the participants were earning more than 100,000 LKR.

Of the 1403 participants 1132 were anti HAV IgG positive. Therefore the overall sero-prevalence of HAV infection was 80.7% (95%CI: 78.64–82.76) for anti HAV IgG. There were 283 (20.2%) individuals under the age group of 14 years or below and out of them, 204 had anti HAV IgG. Therefore sero-prevalence for that age group was 72.1%. The age group 15 years or more comprised of 1120 (79.8%) participants and 928 had anti HAV IgG, therefore sero-prevalence was 82.9% for that age group.

The least percentage of sero-prevalence was observed (66.9%) in the age group of 11–20 years (*n* = 232) followed by 21–30 year group. From the age 31 years upwards, the sero-prevalence exceeded 90%, reaching 100% after 71 years. (Table 1) Urban population showed a sero-prevalence of 83% while females had a sero-prevalence of 82.2% (Table 2). Among different ethnic groups, sero-prevalence of the Sinhalese population was noted as the lowest, i.e. 80.3% (*n* = 1082 out of 1347 Sinhalese). Tamils populations showed 85.7% and Moors were having 100% sero-prevalence.

**Table 1:** Distribution of sero-prevalence according to age groups

Age Group (Years)	Total number of participants	Number IgG positive	Sero-prevalence (%)
1–10	247	174	70.45
11–20	347	232	66.86
21–30	149	104	69.80
31–40	237	221	93.25
41–50	200	184	92.00
51–60	148	142	95.95
61–70	57	57	100.00
71–80	16	16	100.00
81 and above	2	2	100.00
Total	1403	1132	80.7

**Table 2:** Sero-prevalence according to the selected socio demographic characteristics of participants

Characteristics	Total number of participants	Number IgG positive	Sero-prevalence (%)
Sector			
Urban	235	195	83.0
Rural	1168	937	80.2
Sex			
Male	471	366	77.7
Female	932	766	82.2
Ethnicity			
Sinhala	1347	1082	80.3
Tamil	42	36	85.7
Moors	10	10	100.0
Other	4	4	100.0
Total	1403	1132	80.7

Forty two respondents (3.7%) who had sero-positive results (*n* = 1132) claimed that they had viral hepatitis in the past. Out of the total sero- positive cases who claimed that they had viral hepatitis in the past, only 34 (3%) claimed that it was HAV. (Table 3) Past episode of tea color urine was experienced by 14.9% (*n* = 169) and 110 (9.7%) experienced episodes of pale stool during their lifetime out of those who had positive serology (*n* = 1132) results (Table 3). Episode of all three symptoms were experienced by 4.7% (*n* = 53).

**Table 3:** Distribution of individuals who had episodes of clinical symptoms of viral hepatitis in the past (*n* = 1132)

Clinical symptoms experienced in the past	Frequency	Percentage (%)
Yellowish discoloration of eyes	98	8.7
Tea colour urine	169	14.9
Pale color stools	110	9.7
All three symptoms together	53	4.7
Tea colour urine and yellowish discoloration of eyes	64	5.7
Tea colour urine and pale color stools	69	6.1

## Discussion

We found that the overall sero-prevalence of HAV was 80.7%. The sero-prevalence was 72.1% for the age group of 14 years or less and 82.9% for the age group 15 years or more. Urban population showed a sero-prevalence of 83% and for the rural sector it was 80.2%. Females had a sero-prevalence of 82.2% and for males it was 77.7%.

The community-based sero-prevalence survey conducted in the district of Colombo had revealed an overall sero-prevalence of 76.9% in 1975 [9]. In the above study, antibody testing was done by immune adherence hemagglutination test. The age specific as well as sex specific prevalence were also similar in this study and the present study. This shows even after nearly 40 years, the sero-prevalence has not shifted down despite the drastic preventive measures adopted by the health system in Sri Lanka. Therefore it is important to identify risk factor profile of HAV in Sri Lanka, in order to have more focused preventive strategies. Another study conducted in Lady Ridgway hospital, Colombo revealed that 10.4% cases of the children who were admitted to this particular hospital [10]. However, the study was not comparable with the present study as being a hospital-based survey and restricted to a pediatric age group.

Endemicity states are high among Africa, Asia, Central America and South America. Low level of endemicity is present in some of the developed countries such as USA, Canada, New Zealand, Australia, Japan and countries of Northern and Western Europe. But regions like Middle East and Southern and Eastern Europe are categorized under regions with intermediate endemicity [11]. A study conducted in Korea found that overall sero-prevalence of HAV [12] as 63.8%. Anti- HAV IgG test was done using the same ELISA kit as in the present study among 497 randomly selected population [12]. A sero-prevalence survey conducted in Australia revealed an overall 41.1% of sero-positivity [13]. Lagarde conducted a sero-prevalence study in France in 1993 and found 16.3% serology positive cases among military personals [14]. This shows that there are wide variations of sero-positivity even in developed countries. However, sero-epidemiological surveys conducted on children in Pune, India and Egyptian children have reported almost 100% sero-prevalence rates [15]. All most all the countries in the South Asian region can be categorized as high endemic countries [11]. Endemicity of HAV infection depends on the level of sero-prevalence among the specified populations.

Some populations have shown shifting sero-prevalence of HAV infection such as in Chile where an overall prevalence of 54.7% observed in 1990 and 40% in 1998 [16]. Similar finding was observed among the Jordanian children and endemicity was changing towards an intermediate level [17]. Japan has conducted periodical studies in order to find sero-prevalence of HAV and these studies revealed that overall seo-prevalence has dramatically decreased as in 1973 (96.9%), 1984 (96.9%), 1994 (74.3%) and 2003 (12.2%) though susceptibility was increasing annually [18].

Exploring age related sero-prevalence is important to take policy decisions on preventive strategies such as immunization. Some countries have experienced shifting HAV infection to older age groups resulting more symptomatic cases and fatal cases [19]. The present study shows approximately 70% sero-prevalence until 30 years of age. Age group between 31 to 40 years had 93.2% sero-positivity and showed a 20 % increase in sero-positivity compared to the previous age group. Among children and adolescents, relatively low sero-prevalence rates were observed in high income countries in the Asia Pacific region However region rates reached up to 100% sero-positivity after age of 65 years [11].

A study conducted in Western Brazil reveled 16.7% overall sero-prevalence among children ages between 1 and 5 years in 2011 [19]. But there is a marked difference in the current study which determines 70.45% sero-prevalence among age of 1 to 10 years. This high prevalence may be due to disease outbreaks in some parts of the district of Gampaha during 2012 and 2013 period. A study conducted in Jordan showed 26, 32, 44, 63, 78 and 94% sero-prevalence rates in age groups of < 2, 2–4, 5–9, 10–14, 15–19 and > 20 years respectively [17]. Comparatively a low sero-prevalence rate was observed below the age of 9 years which is nearly 40%. It was recommended to initiate a large scale vaccination programs among children based on this finding in Jordan. Nevertheless, according to the present study sero-prevalence has reached a near 70% and hence mass vaccination will not be a supportive strategy.

Some countries have experienced shifting of endemicity due to many reasons including the improvement of environmental hygiene and living conditions [20]. Moreover, the age of acquiring HAV infection is also shifting towards adolescents and adults. This shift leads to a more symptomatic disease, since childhood HAV infection is asymptomatic [19]. Studies conducted in India show heterogeneous exposure to HAV infection and one of the challenges they face is identifying susceptible pockets [20]. Identification of such pockets enables early interventions including vaccinations. However in the present study overall sero-prevalence was equal among the individuals of urban and rural sectors and indicates homogenous exposure in the entire district.

HAV disease is generally asymptomatic. Clinical manifestation of HAV infection is uncommon and around 10 to 25% of the individuals who are infected with HAV are presented with clinical features [21]. These asymptomatic people can transmit the disease in the community. This information would be beneficial in deciding on the policy level interventions. In the present study, it was revealed that only 8.7% had developed clinical jaundice out of those who had positive serology. However, the proportion who developed dark color urine was 14.9% and patients who experienced pale color stools were 9.7%. Out of those who had positive serology, only 3.7% individuals claimed that they had some form of hepatitis infection in the past and 3% claimed that they had HAV infection. This gap between the sero-prevalence and past medical history is most probably due to the unawareness of a previous episode of HAV.

The study has several limitations. This study was conducted in a district of Sri Lanka. To change the policy it is better to consider evidence from a national study. Collected data solely depended on the information given by the participants. Still there is a risk of recall bias specially assessing symptoms and signs. Even though the laboratory adopted several measures of controlling the quality of assessing IgG, the sensitivity and the specificity of the test may vary for a minute change in human behaviour.

## Conclusions

The overall sero-prevalence of HAV was 80.7% among the population of the district of Gampaha. Least percentage of age related sero-prevalence was observed among the age group of 1 to 30 year while it reached to 90% after 31 years and 100% after the age of 70 years. Sero-prevalence of HAV among males (77.7%), females (82.2%), urban population (83%) and rural (80.2%) populations were fairly equal. Periodical serological testing should be done in order to find shifting of endemicity among the population since nearly a 4% increase in sero-positivity can be observed after 42 years. Serological confirmation must be given priority in assessing the burden of disease frequency of HAV.

## Abbreviations

CI: confidence intervals
GND: Grama-Niladari’ Divisions
HAV: Hepatitis A virus
PHNS: Public Health Nursing Sister
SD: Standard deviation
